# The ambiguity of “true” in English, German, and Chinese

**DOI:** 10.1007/s44204-024-00150-1

**Published:** 2024-04-15

**Authors:** Kevin Reuter

**Affiliations:** Institute of Philosophy, University of Zurich, Zürichbergstrasse 43, 8044 Zurich, Switzerland

**Keywords:** Truth, Ambiguity, Cross-linguistic studies, Cross-cultural studies, Pluralism about truth

## Abstract

Through a series of empirical studies involving native speakers of English, German, and Chinese, this paper reveals that the predicate “true” is inherently ambiguous in the empirical domain. Truth statements such as “It is true that Tom is at the party” seem to be ambivalent between two readings. On the first reading, the statement means “Reality is such that Tom is at the party.” On the second reading, the statement means “According to what *X* believes, Tom is at the party.” While there appear to exist some cross-cultural differences in the interpretation of the statements, the overall findings robustly indicate that “true” has multiple meanings in the realm of empirical matters.

## Introduction

Consider the following four statements:

1. It is true that the chemical composition of sugar is $C_{12}$ $H_{22}$ $O_{11}$.
2. It is true that Tom is at the party.
3. It is true that 3 plus 2 equals 5.
4. It is true that it is not illegal for Amanda to cheat on her husband.

Providing an accurate account that captures the intended sense of the phrase “it is true that” across all four types of statements has proven to be a formidable challenge. The correspondence theory, coherence theory, and pragmatic theory of truth, despite their considerable merits, have thus far failed to deliver a comprehensive account.

Proponents of the deflationary theory of truth offer an interesting solution (Ramsey, 1927; Horwich, 1990): they contend that the phrase “it is true that” lacks substantive meaning and can be dispensed without any accompanying loss in semantic meaning. Accordingly, saying “3 plus 2 equals 5” is a short way of saying “It is true that 3 plus 2 equals 5.” However, many scholars remain unconvinced by the deflationary account and continue to explore alternative approaches that might offer a more substantial and satisfactory account of the predicate “true” and the phrase “it is true that.”^1^

The correspondence theory posits that truth is a matter of correspondence between a statement or belief on the one hand, and reality on the other (Russell, 1912; Wittgenstein, 1921). Given this understanding, it follows that one could replace the phrase “it is true that” with the phrase “reality is such that.” And, indeed, the following two statements can be reasonably argued to be equivalent:

1. It is true that the chemical composition of sugar is is $C_{12}$ $H_{22}$ $O_{11}$.
2. Reality is such that the chemical composition of sugar is $C_{12}$ $H_{22}$ $O_{11}$.

However, the statements

3.a Reality is such that 3 plus 2 equals 5.
4.a Reality is such that it is not illegal for Amanda to cheat on her husband.

may not serve as suitable substitutions for (3) and (4). There does not seem to be a tangible reality to assess numerical claims against, in contrast to statements about empirical subject matter (Pederson & Wright 2018). Some scholars have proposed that the notion of truth in relation to (3) and (4) is better understood in terms of coherence with a relevant set of beliefs *X*, rather than as a correspondence with reality (e.g., Putnam, 1981; Young 2001). In light of this view, the coherence theory posits that the phrase “it is true that” can be substituted with “according to belief set *X*,” where the relevant set of beliefs is contextually determined. And, indeed, (3.b) and (4.b) do seem to capture the meaning of “it is true that” for (3) and (4).

3.b According to the laws of arithmetic, 3 plus 2 equals 5.
4.b According to the laws of our country, it is not illegal for Amanda to cheat on her husband.

Despite its intuitive appeal for mathematical and ethical statements, most experts consider the coherence theory to be a less convincing account for statements such as (1) and (2). The reason for this lies in the fact that the truth of a statement concerning the chemical composition of sucrose does not seem to be reliant on any specific set of beliefs or rules, but rather depends on the way in which the world actually is. As we will see below, this expert perspective does not match laypeople’s views on truth.

Some philosophers embrace these complexities and endorse pluralism concerning truth (Wright, 2005; Lynch, 2012). According to *semantic* pluralists, the term “true” has multiple meanings, for instance, “it is true that” could signify “reality is such that” in certain domains and “according to belief set *X*” in others. In regard to the truth conditions of sentences, there are three widely discussed domains: the empirical domain, the mathematical domain, and the ethical/legal domain. Statements (1) and (2) are part of the empirical domain, for which the correspondence theory seems to be better suited, while statements (3) and (4) belong to the mathematical and ethical/legal domains, respectively, for which the coherence theory provides the better criteria (Edwards, 2012). It is worth noting that almost all scholars who advocate semantic pluralism are also domain pluralists (Edwards, 2012), meaning that the truth predicate is ambiguous across different domains only, but not within a single domain.

This paper argues for a more radical claim. Based on new experimental studies, I will argue that “true” is ambiguous within the empirical domain. The studies presented in this paper build on a recent paper by Reuter and Brun (2022), which already suggests that “true” is ambiguous within a single domain. However, Reuter and Brun’s paper has two limitations that are addressed in this paper.

First, the studies conducted in this area have demonstrated that there are two distinct conceptions of truth that individuals utilize in the empirical domain. Nonetheless, these findings do not establish whether there exists any within-subject ambiguity, that is, whether the truth predicate is interpreted differently by the same individuals.^2^ Second, it is essential to exercise caution in drawing conclusions regarding the importance of any evidence supporting the ambiguity of truth. In the absence of cross-linguistic and cross-cultural data, overgeneralization of the results must be avoided. It is plausible that the observed findings are attributable to linguistic and pragmatic effects, which may not hold for other languages. Unless conclusive evidence emerges indicating that this is a widespread phenomenon, the philosophical implications of these results remain limited.

The paper’s approach is as follows: Section 2 reviews previous empirical studies that suggest the term “true” is ambiguous, as well as discusses recent cross-cultural work on the concept of truth. In Section 3, empirical studies will be presented, starting with English, which indicate that “true” is indeed ambiguous within the empirical domain. Section 4 extends these studies to German and Chinese (Mandarin), where the results closely resemble those of the English study. In Section 5, I summarize my findings, provide an account of ambiguity in the empirical domain, and discuss its relevance for recent discussions on the cross-cultural variability of philosophical concepts.

## Related work

### Empirical work on the concept(s) of Truth

Research on the ambiguity of the term “true” has a long and esteemed history. In 1938, Naess conducted extensive interviews with laypeople to ascertain their perspectives on the concept(s) of truth (Naess, 1938). The outcomes have been well documented and discussed in the literature (Barnard & Ulatowski, 2016), and they indicate that people from different backgrounds and perspectives hold diverse conceptions of truth. Naess discovered that every major philosophical account of truth is endorsed by a significant number of laypersons. This finding, while not inherently surprising, demonstrates that each prominent theory of truth enjoys some level of intuitive backing. Thus, the very first empirical studies on people’s thinking about truth already suggest that the term “true” is ambiguous, which several years later was also endorsed by Tarski (1944).

Although Naess’s pioneering work on empirically exploring truth concepts can hardly be faulted for methodological deficiencies, it did not consider the impact of the factor *domain* on his findings. Specifically, when Naess posed questions to his subjects, he did not control for the domain or context that they were considering when responding. It is possible that participants who expressed agreement with the coherence theory were reflecting on examples from the realms of mathematics or ethics, while those who espoused the correspondence theory of truth were reflecting on cases from the empirical domain.

Recent empirical studies on the concepts of truth have been more meticulous in their approach, taking care to account for the influence of the specific domain under consideration. For example, Bernard and Ulatowski (2013) conducted research on differences in agreement with statements such as (T) “If a claim reports how the world is, then it is true.” In their study, some participants were asked to evaluate claims from the mathematical domain, such as “30 + 55 = 85,” while others were presented with claims from the empirical domain, such as “This house is blue.” The results of this study indicate that people more strongly agree with (T) when presented with empirical statements (with a mean score of 3.63 on a 5-point Likert scale) as compared to statements from the mathematical domain (with a mean score of 2.81).^3^

Kölbel (2008) also posits that the term “true” is ambiguous, based on some empirical data he collected from his students. He discovered that a substantial number of his students believed that the statements “Ali G is funny” and “Statements concerning what is funny cannot be true or false” are both true. In this particular example, the ambiguity is not related to a correspondence versus coherence sense of truth but instead to a distinction between a deflationary and a substantial sense of truth, as endorsed by approval of the former and latter statements, respectively.

The results of these studies suggest that there is a plurality of truth concepts that are held by laypeople, but this plurality appears to exist only across domains, not within a single domain. Additionally, while the empirical studies we have examined are certainly significant, they are consistent with pluralistic accounts that have been proposed by scholars like Lynch (2012) and Wright (2005). Therefore, the data provide empirical backing for pluralistic accounts. Consequently, these studies do not challenge established pluralistic accounts.

Regarding the empirical domain, most theorists assume that the correspondence theory of truth is largely supported by the lay concept of truth. Therefore, it would be particularly remarkable to discover the ambiguity of the term “true” for this clearly defined domain. Nevertheless, Reuter and Brun have conducted studies that indicate the term “true” to be ambiguous in the empirical domain. They presented participants with various vignettes, one of which was as follows:

Party: Maria and Peter are students and meet up for a late dinner. Peter asks Maria whether Tom is at the party that they intend to go to after dinner. Maria answers that Tom is at the party. After all, Tom had told her that he would be at the party. When they arrive at the party, it turns out that Tom had changed his plans, and is not at the party.

After reading this vignette, participants were asked the question: “Was Maria’s answer true or false?” If the correspondence theory is correct in stating that the “true” predicate means something along the lines of “the way reality is,” then participants should clearly respond with “false” because Maria’s answer did not correspond with reality. Conversely, if the term “true” is interpreted as “in accordance with the beliefs of the protagonist,” we would anticipate the participants to answer “true.” Through various studies employing different vignettes, Reuter and Brun discovered a distinct division in the population, with roughly 50% of the participants selecting “true” and the other 50% choosing “false.”^4^

Although the results of these studies suggest that the term “true” may be ambiguous even within the empirical domain, it has been rightly pointed out by Kölbel (personal correspondence) that these studies have not established within-subject ambiguity of the truth predicate. It is possible that different individuals may have distinct notions of what “truth” means. Thus, one group of people may consider “true” to refer to correspondence with reality, while another group may interpret it in a more coherentist manner. If this were the case, individuals would not recognize the ambiguity of the term but rather apply it consistently based on their understanding of its meaning.

To address this challenge and establish the ambiguity of “true” within the empirical domain, I conducted several new empirical studies that are presented in Sections 3 and 4.

### Cross-cultural perspectives on Truth

The concept of truth has traditionally been regarded as universal among citizens of the world, particularly by philosophers who have seldom questioned the representativeness of the Anglo-American or at least Western perspective on such concepts. However, this perception has shifted considerably in recent years, with empirical research demonstrating that non-Westerners may think quite differently about reference, free will, and other concepts (Ahlenius and Tännsjö, 2012; Machery et al., 2015; Robbins et al., 2017; Hannikainen et al., 2019). On the other hand, various studies exist that indicate little cross-cultural variation for many philosophical concepts (Kim et al., 2016; Rose et al., 2020; Yang et al., 2021; Kim & Yuan, 2019), sparking a lively debate on the universality and uniformity of philosophical concepts involving among others Knobe (2019) and Stich and Machery (2022).

Some philosophers, such as Goldman (1999), explicitly endorse the uniformity of truth across cultures. Maffie (2001) goes as far as stating that “the majority of 20th-century Anglo-American epistemologists from William Alston and Roderick Chisholm to Bertrand Russell and Barry Stroud” (p. 267) believe truth to be more or less similar across the globe. However, anthropological research paints a rather different picture. Based on his own research, Maffie (2002) argues that the indigenous Nahuatl-speaking peoples of the High Central Plateau of Mexico lack a concept of truth. Scharfstein (2001) claims that no single conception of truth is shared universally, building on research on Confucian, Inuit, Navajo, and Taoist (among others) conceptions. Hall (2001) argues that Chinese conceptions of truth—in contrast to Western conceptions—are often concerned with self-actualization and authenticity. McLeod (2011) adds to this that within classical Chinese philosophical frameworks, one often sees pluralist conceptions in action. And Wyatt (2018) draws attention to Wiredu (1985)’s work on the notion of truth in the Ghanaian language Akan, which differs in detail from the English conception of truth.

**Fig. 1:**
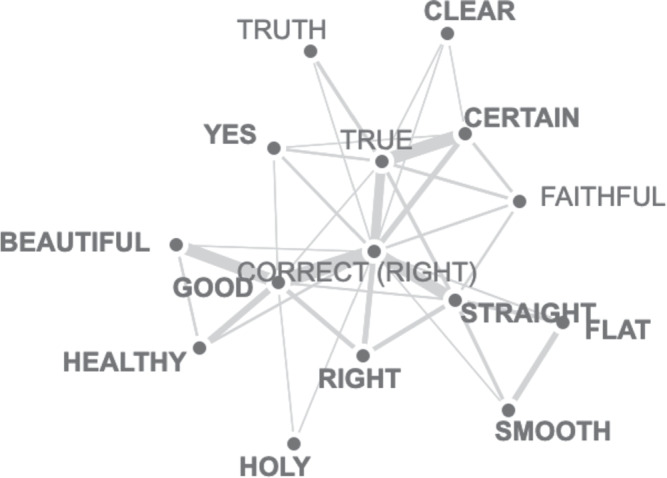
Colexification subgraph of the concept true

Further evidence that the concept of truth is not uniform across the globe comes from colexification data (see https://clics.clld.org). Figure 1 displays the colexification network of the concept true. Strong links between two concepts in colexification graphs demonstrate that in many languages, both concepts are expressed with the same word. The subgraph for true shows very strong connections between true and certain, as well as between true and correct. Further links exist between true and clear, faithful, and straight. To take just one example to illustrate the close link between true and certain: According to the clics3 subgraph for true, there are 38 languages (including Austroasiatic, Indo-European, Mayan, Sino-Tibetan, Turkic, and Uralic languages) which have a single word only to express both certain and true. Although this does not rule out decisively the possibility that these languages feature ways for “true” to express correspondence with reality, it appears more plausible that “true” in these languages expresses coherence with existing beliefs that makes a claim certain.

Despite the rich anthropological research on the concept of truth, there is very little empirical data on laypeople’s use of the truth predicate in languages other than English. One remarkable and very recent exception is Mizumoto’s cross-linguistic work on English and Japanese truth predicates (2022). The collective data imply that moral-political factors exert a stronger influence on the usage of truth predicates in Japanese compared to English. Although the results of Mizumoto’s empirical studies are not central for the studies presented in this paper, I fully agree with Mizumoto’s call for more cross-linguistic and cross-cultural research on the concept(s) of truth. So, let us answer his call.

## Study 1: “true” in English

The primary objective of Study 1 is to provide clear evidence in support of the inherent ambiguity of the term “true” within the empirical domain. While prior research (cf. Reuter & Brun, 2022) has established the presence of differing truth concepts held by distinct individuals, they did not investigate the degree to which ambiguity is present *within subjects*. Specifically, they did not explore whether individuals recognize and apply multiple truth predicates, as discussed earlier. The present study aims to fill this critical gap in the literature by examining the extent of ambiguity inherent in individual truth conceptions within the empirical domain.

To accomplish this objective, I transitioned from a between-subjects design—wherein participants respond to a truth question regarding a single case—to a within-subjects design, whereby participants encounter two distinct cases. The rationale behind this methodological shift is to assess whether “true” indeed embodies inherent ambiguity. Specifically, I hypothesized that if such ambiguity exists, a substantial proportion of individuals will provide correspondentist responses to one case and coherentist responses to the other.

Initial exploratory investigations indicated that a significant majority of individuals tend to provide correspondentist responses when responding to truth inquiries for *scientific matters* such as the chemical composition of molecules. Thus, I formulated experimental scenarios that differed minimally but were discernible by their topic, namely empirical-scientific versus empirical-mundane topics.

### Methods

The following two scenarios were presented to participants in randomized order.

Party: Maria and Peter are students and meet up for a late dinner. Peter asks Maria whether Tom is at the party that they intend to go to after dinner. Maria answers that Tom is at the party. After all, Tom had told her that he would be at the party. When they arrive at the party, it turns out that Tom had changed his plans, and is not at the party.
Chem: Jennifer and Paul are students and meet up for a late dinner. Paul asks Jennifer what the chemical composition of sugar is. Jennifer answers that the chemical composition of sugar is C12 H18 O9. After all, her father had told her that this is the chemical composition of sugar. When they discuss the composition of sugar in their chemistry class the next day, it turns out that the chemical composition of sugar is C12 H18 O11.

As evident from the presented material, the two scenarios are comparable in terms of their structure and content. The primary distinction between them is the topic of discourse, namely the presence of Tom at a party in one case and the chemical composition of sugar in the other. In both scenarios, the central protagonist (Maria/Jennifer) provides a response that is congruent with their relevant beliefs at the time of inquiry. Maria affirms the presence of Tom at the party based on what he had told her beforehand. Jennifer imparts information regarding the chemical composition of sugar as relayed by her father. Nonetheless, in both cases, the responses do not correspond to the objective state of the world at the time of inquiry. Consequently, the scenarios embody a situation in which responses align with relevant beliefs but do not accurately reflect reality.

In order to avoid confounding effects due to the nature of the specific case, I also wrote two further vignettes, which were also presented to a different set of participants in randomized order.

Tech: Maria is a scientist working for a university. She has recently conducted an experiment for which she bought a new technical device. One morning, her boss Lucy asks her whether Maria can show her the technical device. Maria answers that the technical device is in the laboratory. When Maria goes to the laboratory, she finds out that a burglar has stolen several objects over night, among them the new device.^5^
Gala: Clara is a scientist working for a university. She has recently conducted an experiment to determine the distance between Earth and the Sagittarius Galaxy. One morning, her boss Amanda asks her whether Clara can tell her what the main finding of the experiment is. Clara answers that the Sagittarius Galaxy is 30,000 light years away. When Clara looks carefully at the experiment and data a little later, she finds out that a lab assistant has manipulated the experiment and that the Sagittarius Galaxy is 70,000 light years away.

Again, each participant was assigned to both scenarios with randomized order. The following questions were given to participants after the respective vignettes:

Party: Was Maria’s answer true or false?
Chem: Was Jennifer’s answer true or false?
Tech: Was Maria’s answer true or false?
Gala: Was Clara’s answer true or false?

People were presented with two options: (1) true and (2) false. While a “True” response implies that the participant subscribes to coherentism, at least with regard to the scenario in question, a “False” response suggests that the participant applies a correspondentist notion of truth. Eighty participants, native English speakers from the UK and the USA, were recruited via Prolific Academic and assigned to the Party and Chem cases. One participant was excluded for not having answered the main truth questions. Of the remaining 79 participants with an average age of $M_{age} = 37.9$ years, 40 identified as female and 37 as male. For the Tech and Gala cases, 80 participants (38 female, 40 male, 2 other) with a mean age of $M_{age} = 38.4$ (native English speakers from the UK and the USA) were recruited via Prolific. The data files are available via the Open Science Framework.

**Fig. 2:**
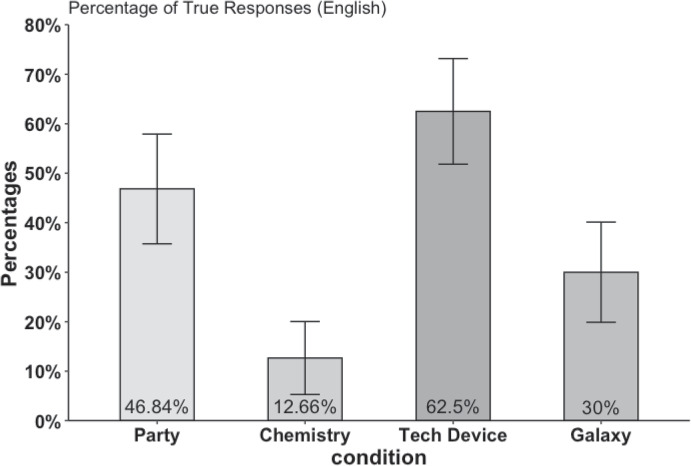
Percentages of true responses to the scenarios of Study 1. Error bars indicate 95% confidence intervals

#### Results

Figure 2 displays the percentage of “true” responses for all four scenarios. In the scientific scenario Chemistry, only a small minority of participants, 12.66% (95% CI (5.28%, 20.04%)), believe Jennifer’s answer to be true, suggesting that most people apply a correspondentist notion of truth. The situation looks different in the more mundane scenario: In the party case, 46.84% (95% CI (35.76%, 57.91%)) of participants believe Maria’s answer to be true. Comparing the two conditions using a paired *t*-test, there is a significant difference in the proportion of “true” responses between the Chemistry and Party conditions ($t = 5.750, p < 0.001$) with an effect size of 0.80 (Cohen’s *d*). Importantly, 36.7% of the participants responded that Maria’s answer was true in the Party case, and Jennifer’s answer was false in the Chemistry case. Regarding order effects, for Chemistry, there is no significant order effect ($t(49) = 1.33, p = 0.1876$), indicating that the order of presentation does not significantly impact the proportion of “true” responses. However, for Party, there is a significant order effect ($t(49) = -2.30, p = 0.0239$), suggesting that the order in which the “true” statement in the Party case was answered does influence the proportion of TRUE responses. Participants are more likely to respond “true” when the Party vignette is presented first.

In the second set of conditions, similar results were obtained in regard to the difference between the two cases. In the scientific scenario (Galaxy), 30.00% (95% CI (19.89%, 40.11%)) of participants considered the statement true. In contrast, a substantial majority of participants in the Tech Device case, about 62.50% (95% CI (51.82%, 73.18%)), perceived the statement as true, indicating a tendency towards a coherentist theory of truth. A comparative analysis using an independent *t*-test reveals a statistically significant difference in the proportion of “true” responses between the “Tech Device” and “Galaxy” conditions ($t = 5.324, p < 0.001$, Cohen’s *d* = 0.685), demonstrating that the subject matter significantly affects participants’ truth judgements. Crucially, 36.3% of the participants responded that Maria’s answer was true in the Tech Device scenario, and Clara’s answer was false in the Galaxy scenario. As for the effect of presentation order, a two-tailed independent *t*-test turned out not to be significant ($t = 0.876, p = 0.384$).

#### Discussion

Study 1 provides strong evidence that the predicate “true” is ambiguous in the empirical domain. Fifty-eight out of 159 adhered to two distinct notions of truth, despite the structural identity of the statements pertaining to empirical matters. The main divergence between these statements was the specific content of the information conveyed. In the Party and Technical Device conditions, the responses of the protagonists pertained to ordinary facets of life (i.e., the location of Tom and a technical device, respectively), whereas the responses in the Chemistry and Galaxy conditions revolved around scientific aspects of life (i.e., the chemical composition of table sugar and the distance between Earth and the Sagittarius Galaxy).

## Study 2: “*Truth*” in German and Mandarin

The outcomes of Study 1 provide compelling evidence for the ambiguity of the truth predicate in the empirical domain. To strengthen the conclusion that “true” is indeed ambiguous, it would be advisable to demonstrate that the findings are not specific to the English language but rather consistent across diverse languages and cultures. Failure to replicate the results in other linguistic and cultural contexts may prompt critics to proffer an error theory that attributes the English laypeople’s response patterns to a mere linguistic or pragmatic aspect of the term “true,” rather than a genuine conceptual understanding of truth.

In addition to providing further support for the ambiguity of the term “true,” another reason for exploring individuals’ responses in other linguistic and cultural settings is the growing awareness within academia that philosophical concepts, as understood in Western cultures, may not be universally applicable (e.g., the Geography of Philosophy project ). Thus, it is recommended to examine fundamental philosophical ideas from a cross-linguistic and cross-cultural perspective. To this end, Study 2a reports the outcomes of an investigation conducted in German, while Study 2b examines truth predicates among Mandarin speakers.

### Study 2a: *Truth* in German

#### Methods

In the Chemistry case, only very few participants in Study 1 gave a “true” response. I therefore decided to continue with and focus on the Party and Chemistry case. Certainly, future studies should extend the range of cases to provide more robust evidence. The Party and the Chemistry cases were translated by the author who is a native German speaker. The scenarios that were presented to 80 native German participants ($M_{age} = 37.9$ years, 40 female, 39 male, 1 other, all recruited on Prolific) read as follows:

Party: Maria und Peter sind Studenten und treffen sich zu einem späten Abendessen. Peter fragt Maria, ob Tom auf der Party ist, zu der sie nach dem Essen gehen wollen. Maria antwortet, dass Tom auf der Party ist. Immerhin hatte Tom ihr gesagt, dass er auf der Party sein würde. Als sie auf der Party ankommen, stellt sich heraus, dass Tom seine Pläne geändert hat und nicht auf der Party ist.
Chem: Jennifer und Paul sind Studenten und treffen sich zu einem späten Abendessen. Paul fragt Jennifer, wie die chemische Zusammensetzung von Zucker ist. Jennifer antwortet, die chemische Zusammensetzung von Zucker sei C12 H18 O9. Schließflich hatte ihr Vater ihr gesagt, dass dies die chemische Zusammensetzung von Zucker ist. Als sie am nächsten Tag im Chemieunterricht über die Zusammensetzung von Zucker sprechen, stellt sich heraus, dass die chemische Zusammensetzung von Zucker C12 H18 O11 ist.

Each participant was assigned to both scenarios in randomized order. The following questions were given to participants after the respective vignettes, after which they were presented with two options (1) Wahr. (2) Falsch:

Party: War Marias Antwort wahr oder falsch?
Chem: War Jennifers Antwort wahr oder falsch?

#### Results

**Fig. 3:**
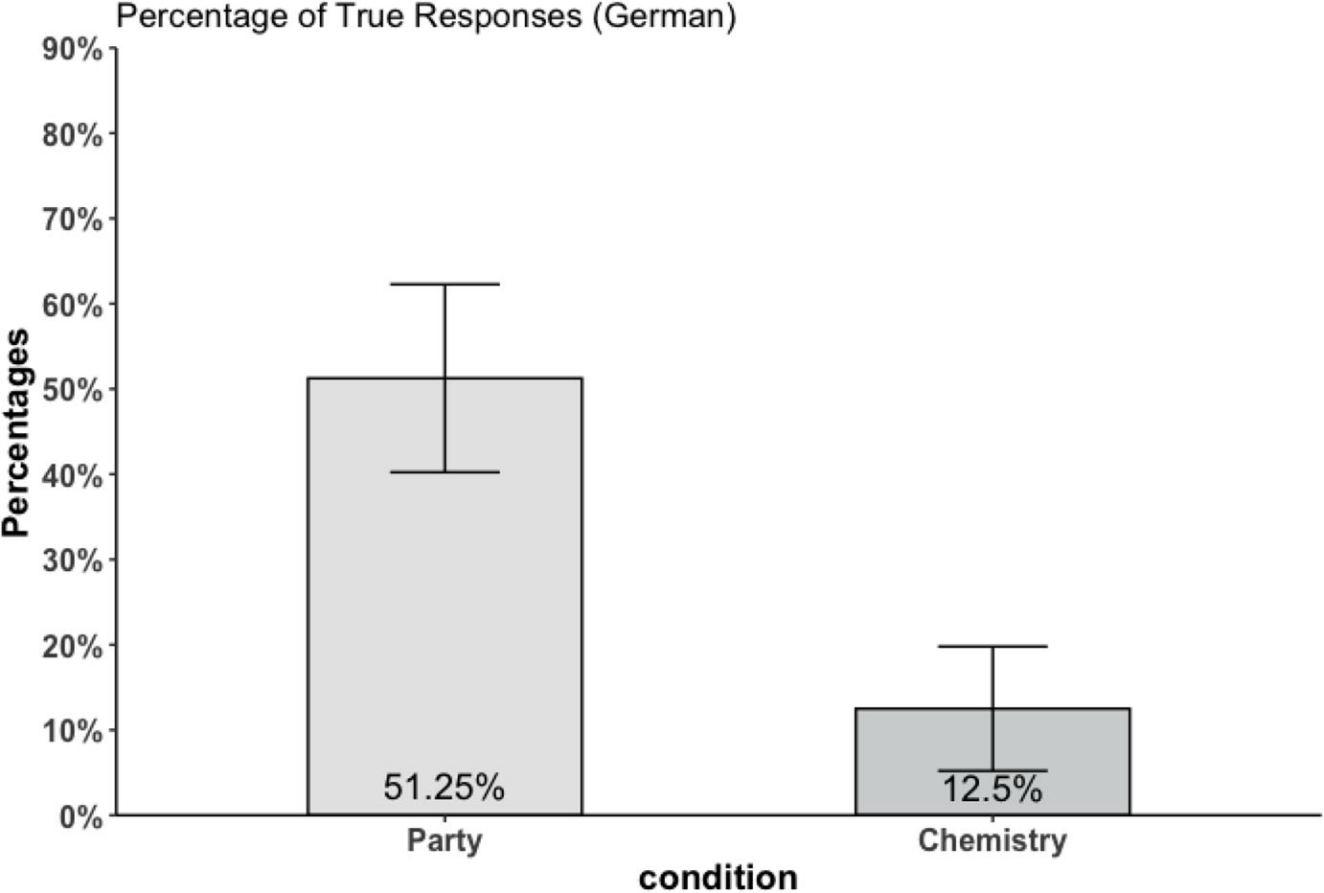
Percentage of true responses to the scenarios of Study 2a. Error bars indicate 95% confidence intervals

The results (see Fig. 3) of the Party and Chemistry condition in German reveal a similar pattern to the results in English. While only a small minority of the participants think that Jennifer’s answer in the Chemistry scenario is true, 12.50% (95% CI (5.21%, 19.79%)), participants were split in the Party case with 51.25% (95% CI (40.23%, 62.27%)) answering “true.” The difference between both conditions was highly significant ($t = 6.163, p < 0.001$), and a large effect size Cohen’s d of 0.908. 34 participants selected “wahr” in the Party case and “falsch” in the Chemistry case, indicating that *true* is ambiguous not just in English but also in German. No significant order effect of the within-subject design was determined ($t = 1.299, p = 0.198$).

### Study 2b: *Truth* in Mandarin

The results of Study 2a provide evidence that the ambiguity of the predicate “true” that we recorded in Study 1 is not an artifact of the English language. Still, German and English are not only strongly related Indo-European languages, most native speakers of these two languages belong to the same Western culture. Stronger evidence for cross-cultural ambiguity of truth predicates needs to involve speakers of non-Western languages. In the following study, I tested the responses of Mandarin speakers to the Party and Chemistry vignette.

### Methods

The two vignettes were initially translated using the DeepL translator. I then consulted with a Mandarin speaker who is also a Chinese university teacher about the translations. She made several suggestions for improving the Chinese vignettes.^6^ The two scenarios that were presented to 79 native Mandarin speakers^7^ ($M_{age} = 33.1$, 39 female, 39 male, 1 other, all participants recruited on Prolifc) read as follows:

Party
 
Chem
 

As with Study 1 and Study 2a, each participant was assigned to both scenarios in randomized order. The following questions were given to participants after the respective vignettes:

Party
 
Chem
 

Participants were presented with a binary choice between two response options: (1)  (2)  While  is one possible Mandarin expression for the English term “true,” there are other equivalent Chinese expressions that include  , and others that may be translated as “true,” “real,” and “correct,” depending on the context. The selection of the response options  and  was based on their high frequency of usage and naturalness in conveying the idea that a statement is true.

#### Results

**Fig. 4:**
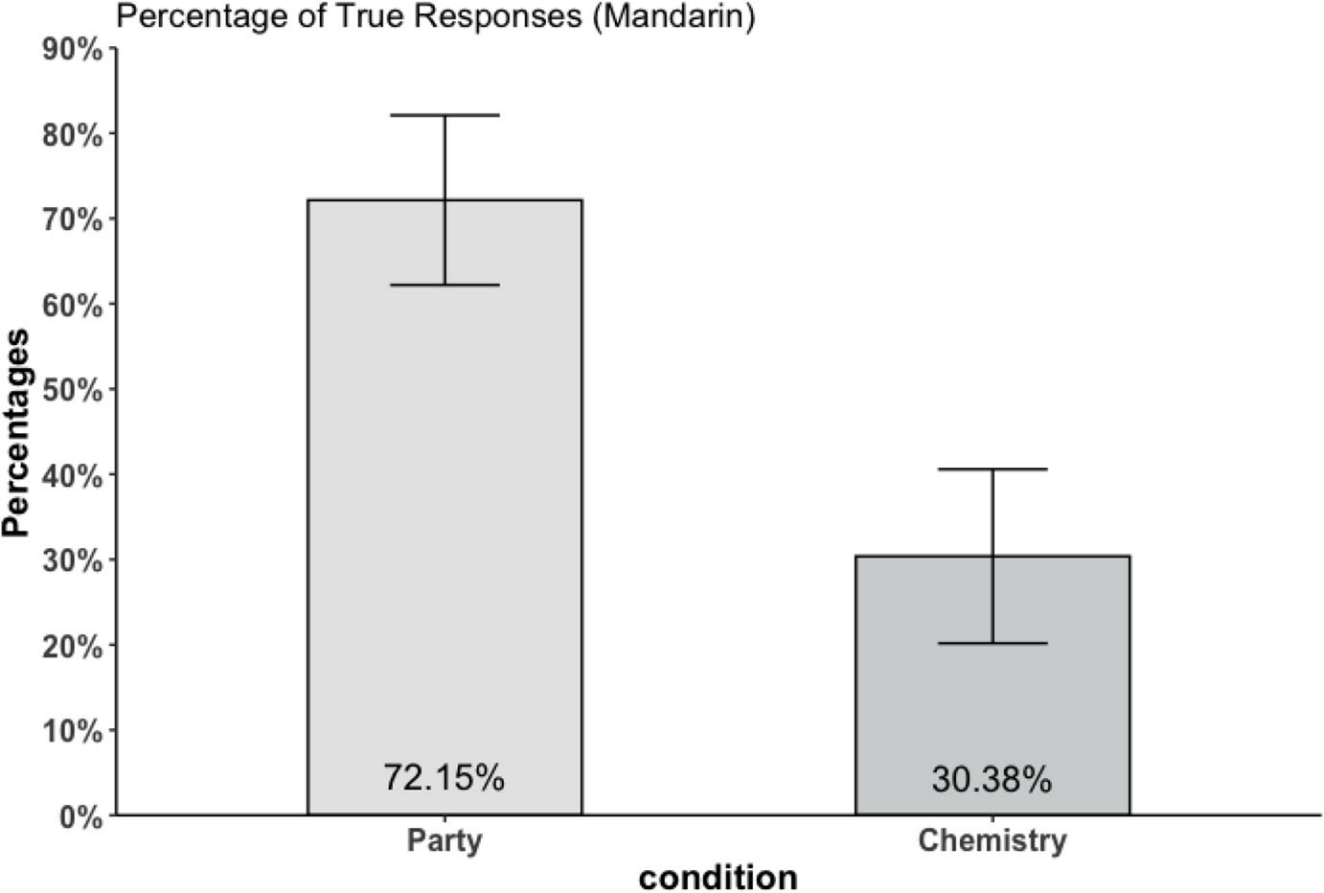
Percentage of true responses to the scenarios of Study 2b. Error bars indicate 95% confidence intervals

In Fig. 4, the percentages of true responses for both the Party and Chemistry vignettes are illustrated. Consistent with the findings for the English and German vignettes, there is a pronounced disparity in the proportion of true responses for the Party case (72.15%; 95% CI (62.20%, 82.10%)) and Chemistry case (30.38%; CI (20.17%, 40.59%)). The difference observed in true responses between the scenarios is 41.77%, akin to that observed in the previous two studies. Moreover, the discrepancy between the two conditions was statistically significant, with a paired *t*-test yielding a *t*-value of 6.806 ($p < 0.001$) and a large effect size (Cohen’s d = 0.914). Thirty-seven participants chose “false” in the Chemistry case and “true” in the Party case. Notably, the overall proportion of true responses was higher compared to the responses of English and German participants.^8^ This pattern may reflect a greater emphasis on authenticity within the Chinese concept of truth, as has been suggested by Hall (2001).

## General discussion

### Summary of the results

The empirical studies were designed to test whether and when the term “true” is ambiguous within the empirical domain. Let us begin by interpreting people’s responses to the chemical scenario. We started our discussion wondering what the correct interpretation of “it is true that” would be in various cases. For statement (1), we considered interpretations along the lines of (1.a) and (1.b):

1. It is true that the chemical composition of sugar is $C_{12}$ $H_{22}$ $O_{11}$.
2. Reality is such that the chemical composition of sugar is $C_{12}$ $H_{22}$ $O_{11}$.
3. According to belief set $X_c$, the chemical composition of sugar is $C_{12}$ $H_{22}$ $O_{11}$.

The belief set $X_c$ we considered in the Chemistry condition consists of one explicit and one implicit belief. The explicit belief is as follows: (i) Jennifer’s father stated that the chemical composition of sugar is $C_{12}$ $H_{22}$ $O_{9}$. The implicit belief is as follows: (ii) Jennifer’s father is a reliable source of information on school chemistry. Jennifer’s claim that the chemical composition of sugar is $C_{12}$ $H_{22}$ $O_{9}$ is a claim that is coherent with these two beliefs, i.e., with belief set $X_c$. However, that very claim does not correspond with reality. As such, our design was able to pit two competing readings of the truth predicate against each other.

The results clearly favor a correspondence interpretation. Across the languages English, German, and Mandarin, only a relatively small fraction of participants provided answers that are *not* in line with the correspondence theory, and thus, (1.a) seems to be the correct interpretation of (1).

Our second case, the party scenario, examined possible interpretations for (2), namely, (2.a) and (2.b):

2 It is true that Tom is at the party.
2.a Reality is such that Tom is at the party.
2.b According to belief set $X_p$, Tom is at the party.

Analogous to the chemical case, $X_p$ consists of an explicit belief and an implicit belief. The explicit belief is as follows: (i) Tom stated that he would be at the party. The implicit belief is as follows: (ii) Tom is a reliable source of information on Tom’s whereabouts. Maria’s claim that Tom is at the party is a claim that is coherent with these two beliefs, or, in other words, with belief set $X_p$. Nevertheless, that very claim does not correspond with reality. Again, our design was supposed to pit two competing theories against each other.

For all three languages we tested, i.e., English, German, and Mandarin, we found a split in the population. While some people answered along the lines of the correspondence theory, a substantial amount of participants responded in a way that fits a coherentist interpretation, expressed by (2.b).

Importantly, by using a within-subject design—participants were given both the chemistry as well as the party case—we now know that almost 40% of the participants responded by saying that Jennifer’s answer about the chemical composition was false, but Maria’s answer about Tom’s whereabouts was true. Jennifer’s answer was coherent with her belief set $X_c$ but failed to correspond with reality. Maria’s answer was coherent with her belief set $X_p$ but failed to correspond with reality. This suggests that these participants think (1.a) to be a correct interpretation of (1) and (2.b) to be a correct interpretation of (2). Therefore, the results indicate that “it is true that” is ambiguous between “reality is such that” and “according to belief set *X*.”

If my interpretation of the empirical results is correct, we have established ambiguity of the truth predicate in the empirical domain. But how can we explain the results of the empirical studies?

### Explaining the ambiguity of “true”

Studies 1, 2a, and 2b suggest the truth predicate to be ambiguous between two readings. So far, however, we lack an understanding of how people disambiguate between a correspondence and a coherentist interpretation of the truth predicate. Several factors are plausible influences on the disambiguation process which I will discuss in turn. These are stakes, reliability, and domain.

#### Stakes

Although all scenarios describe fairly common conversations between friends, readers might consider the stakes of the cases to vary considerably. In the party case, Maria tells her friend Robert that Tom is at the party. Arguably, not much hinges on whether Tom is at the party or not because Maria and Robert wanted to go to the party anyway. Hence, the stakes are relatively low. In contrast, one might think that telling a friend the chemical composition of a molecule might have rather important downstream consequences like passing or failing an exam. If stakes were indeed an important factor, then raising the stakes in the party scenario or lowering the stakes in the chemical scenario should change people’s judgements about the truth of the statement in question.^9^

#### Reliability

I have argued that both belief sets, i.e., $X_c$ and $X_p$, contain an implicit belief about the reliability of the informant. The father of Jennifer is a reliable source on school chemistry, and Tom is a reliable source about his whereabouts and future plans. The perception of reliability might vary, however, considerably in these two cases. There is no human source more reliable—assuming honest agents—about a subject’s whereabouts and future plans than the very subject themselves. The same cannot be said for Jennifer’s father. Unless he is an expert on the chemistry of sugar, Jennifer could and perhaps should have consulted a different source of information than her father when it comes to the chemical composition of sugar, e.g., her chemistry textbook. If the reliability of the information source were a decisive factor, then “making” Jennifer’s father a chemistry expert, or asking not Tom directly, but rather another friend of Tom, should likely influence which reading of “true” people adopt.

#### Domain

Statements (1) and (2) clearly belong to the empirical domain: It is an empirical fact of the matter whether Tom is at the party and an empirical fact of the matter whether the chemical composition of sucrose (table sugar) is $C_{12}$ $H_{22}$ $O_{11}$. For this very reason, I argued that the results indicate within-domain ambiguity of the term “true.” One might object, however, that we need a more fine-grained taxonomy of domains. Whereas it is an empirical matter of fact that Tom went to the party, it is certainly not a quantifiable and reproducible scientific fact. It could have been the case that Tom was at the party. In contrast, it could not have been the case that sugar is $C_{12}$ $H_{22}$ $O_{11}$ (unless we contemplate nearby possible worlds).^10^

Why would any of these three factors have an influence on which sense of “true” people select when talking about truth? While we have largely bracketed the pragmatic theory of truth from our discussions, it seems that pragmatic aspects might well be the key to understand how people disambiguate the truth predicate in the empirical domain. The pragmatic theory of truth takes the meaning of the phrase “it is true that” to be roughly equivalent to the phrase “it is useful to believe that.” While I do not hold that the pragmatic theory gives us a convincing account of the meaning of the concept of truth, pragmatic aspects might well have a role to play in the disambiguation between a correspondence and a coherentist reading of “true.” Let me explain:

If the stakes are high, it is, arguably, more useful to believe a proposition that corresponds with reality than to believe a proposition that is coherent with a set of beliefs. Consider the case in which a doctor needs to decide whether she conducts a difficult and risky heart operation. It is not very useful to believe the evidence that is consistent with the patient having a heart problem if that patient’s heart functions normally. It is, however, more useful to believe such evidence, if the doctor *merely* prescribes medicine that would benefit the patient in case he has a heart problem, but would not harm him. Note that I do not argue that pragmatic considerations determine that we interpret “it is true that” as being equivalent to “it is useful to believe that.” Rather, I argue that these pragmatic considerations influence whether people interpret “it is true that” in a correspondence or coherentist manner. The higher the stakes, the more useful it is to believe in matters of fact and thus to interpret “it is true that” to mean “reality is such that.”

Similar considerations apply to the reliability of the source of information. In most circumstances, it is useful to believe highly reliable sources and to refrain from believing unreliable ones. It seems prudent to believe Tom when he tells me that he will go to a party even if there is a likelihood that he will change his mind. It seems less prudent to believe my father on the chemical composition of sugar unless I have very good reasons to trust his judgement. Again, I do not think that the usefulness of believing reliable sources of information favors the pragmatic theory of truth, but rather that the usefulness may well have a role to play in disambiguating whether a person entertains a correspondence or a coherentist reading of “true.”

It appears reasonable to suggest that a combination of these three elements—stakes, reliability, and domain—may influence individuals’ judgements of truth. A reviewer for this journal has expressed a reservation that these elements could directly sway the study’s outcomes, bypassing truth judgements altogether. This reservation resonates with the findings of Reuter and Brun (2022), who explored various versions of the substitution objection. Their investigation encompassed the possibility that participants might interpret questions of truth as pertaining to issues of (a) truthfulness, (b) the appropriateness of giving certain answers (assertion norms), (c) the possession of adequate epistemic justification, and (d) the idea of something being “true for” a person. Their research suggests with considerable confidence that participants do not substitute a question of truth with a related question. That said, more studies are, of course, advisable to rule out further versions of the substitution objection.

### Cross-linguistic and cross-cultural differences

The cross-linguistic and cross-cultural studies of this paper have two purposes. First, they aim to investigate the robustness of the ambiguity of the truth predicate in the empirical domain. Without such studies, not only are we limited in making more general claims about the concept of truth, but we also invite the objection that the purported ambiguity of the truth predicate might be a linguistic-cum-pragmatic aspect of the English language. Second, finding cross-linguistic and cross-cultural similarities and differences is a viable research endeavor in its own right and particularly fascinating when it comes to a concept like truth that plays such a vital role in science and society.

In recent years, a debate has arisen regarding whether experimental philosophical research conducted over the last two decades has shown that philosophical concepts are generally stable and uniform across languages and cultures, or whether the research has highlighted significant differences that require rethinking claims of universality and uniformity. While Knobe (2019) recognizes that some studies have uncovered cross-linguistic and cross-cultural variations, he places greater emphasis on the stability and robustness of various factors and features of concepts in people’s thinking. In contrast, Stich and Machery (2022) disagree with Knobe’s position and contend that the substantial amount of cross-linguistic and cross-cultural differences underscore the need to “come to grips with the problems that result when the concepts of marginalized people, and the world views that incorporate those concepts are ignored” (p. 33).

The cross-cultural findings presented in this paper contribute to both sides of the ongoing debate. The results suggest that the overall pattern of differences between the Party and Chemistry case does not exhibit substantial variation across English, German, and Chinese native speakers. Thus, the ambiguity of the truth predicate appears to be culturally stable in these scenarios. However, the data also indicates that the proportion of true responses across both conditions is considerably higher for Chinese native speakers than for English and German speakers. Although caution must be exercised in drawing conclusions from a limited number of vignettes, a closer examination of these variations could provide intriguing insights into differences in truth conceptions between Western and East Asian cultures.

## Concluding remarks

Semantic pluralism regarding truth is widely discussed both in philosophy and anthropology. Much of this discourse assumes that the predicate “true” is ambiguous only *across* domains. However, this paper argues for *within-domain* ambiguity of truth. Through a series of studies conducted with English, German, and Mandarin speakers, we have compelling reasons to adopt a much more intricate understanding of the various meanings of “true.” In the case of commonplace statements such as “Tom is at the party,” some individuals evaluate its truth based on whether the statement accurately corresponds to reality, while others assess its truth based on whether a coherent set of beliefs supports the statement.

The ambiguity of truth in the empirical domain can have far-reaching consequences, particularly in terms of the risk of miscommunication, whether intentional or unintentional. Reuter and Brun (2022) argue for conceptually re-engineering truth in a way that minimizes the likelihood of such miscommunication.^11^ The results of this paper offer a promising approach for establishing common ground among individuals by disambiguating truth talk. Notably, the majority of English, German, and Mandarin participants surveyed applied a correspondence notion of truth to scientific statements. This allows individuals to reconcile differences in assessing whether statements are true, as they can evaluate whether they view truth through scientific guidelines or whether they favor coherence over correspondence.

## Data Availability

The empirical data supporting the findings of our studies can be found hosted on the Open Science Framework (OSF). For full access to the datasets, please visit the following link: https://osf.io/5b68z/.

## Footnotes

1 While metaphysical claims about truth can be distinguished from claims about the semantics of the concept truth and the predicate “true,” for the purposes of this paper, I take a Tarskian approach, according to which different theories of truth provide different definitions of the concept of truth (Tarski, 1944). For a critical perspective, see Wyatt (2016).

2 Reuter and Brun (2022) have only conducted between-subject experiments, i.e., each participant in their studies was presented with only one case.

3 Ulatowski (2017) offers a comprehensive argument in support of truth pluralism, drawing upon numerous empirical studies concerning the popular understanding of truth.

4 Readers who are not familiar with these results might object that laypeople might have misinterpreted the truth question as a truthfulness question, i.e., they only wanted to communicate that the protagonist was not telling any lies. Alternatively, subjects might have been applying a subjective notion of truth, according to which a statement can be true *for* one person and false *for* another. By running additional studies as well as analyzing qualitative responses, Reuter and Brun provide considerable evidence against these objections.

5 This scenario was adopted from Reuter and Brössel (2018).

6 I would like to thank Ai-Linh Achermann for her support in translating the vignettes into Mandarin as well as discussing the various options of translating the predicate “true.”

7 One participant was excluded for not answering the test questions. Please note that the participants in this study are native Chinese speakers but do not live in China. Through Prolific, one can only recruit Chinese speakers from outside of China.

8 No statistical comparison between response patterns from English, German, and Chinese responses was conducted given that these studies were done at different points in time.

9 Arguably, the stakes of telling your boss what the main experimental finding of an experiment is are also more important than telling her where a technical device is. Thus, the Tech Device and Galaxy cases do not help us decide whether stakes are an important factor in the disambiguation process.

10 Relatedly, the content at issue in the party case was an event that happened at a specific time and place. The content at issue in the chemical case is a general property of a molecule type.

11 For a similar approach to engineering conspiracy theory, see Napolitano and Reuter (2021) as well as Reuter and Baumgartner (forthcoming).
